# *Fusobacterium nucleatum* Activates Endoplasmic Reticulum Stress to Promote Crohn’s Disease Development *via* the Upregulation of CARD3 Expression

**DOI:** 10.3389/fphar.2020.00106

**Published:** 2020-02-21

**Authors:** Pan Cao, Yongyu Chen, Xufeng Guo, Yan Chen, Wenhao Su, Na Zhan, Weiguo Dong

**Affiliations:** ^1^ Department of Gastroenterology, Renmin Hospital of Wuhan University, Wuhan, China; ^2^ Key Laboratory of Hubei Province for Digestive System Disease, Renmin Hospital of Wuhan University, Wuhan, China; ^3^ Central Laboratory, Renmin Hospital of Wuhan University, Wuhan, China

**Keywords:** *Fusobacterium nucleatum*, intestinal mucosal barrier, endoplasmic reticulum stress, Crohn’s disease, gene regulation

## Abstract

There is increasing evidence that members of the gut microbiota, especially *Fusobacterium nucleatum* (*F. nucleatum*), are associated with Crohn’s disease (CD), but the specific mechanism by which *F. nucleatum* promotes CD development is unclear. Here, we first examined the abundance of *F. nucleatum* and its effects on CD disease activity and explored whether *F. nucleatum* aggravated intestinal inflammation and promoted intestinal mucosal barrier damage *in vitro* and *in vivo*. Our data showed that *F. nucleatum* was enriched in 41.21% of CD tissues from patients and was correlated with the clinical course, clinical activity, and refractory behavior of CD *(P* < 0.05). In addition, we found that *F. nucleatum* infection is involved in activating the endoplasmic reticulum stress (ERS) pathway during CD development to promote intestinal mucosal barrier destruction. Mechanistically, *F. nucleatum* targeted caspase activation and recruitment domain 3 (CARD3) to activate the ERS pathway and promote *F. nucleatum*-mediated mucosal barrier damage *in vivo* and *in vitro*. Thus, *F. nucleatum* coordinates a molecular network involving CARD3 and ERS to control the CD process. Measuring and targeting *F. nucleatum* and its associated pathways will provide valuable insight into the prevention and treatment of CD.

## Introduction

Crohn’s disease (CD) is a chronic and disabling disease that can seriously affect quality of life. The natural history of CD can lead to intestinal damage, such as stenosis, fistula, or bowel resection (Malluta et al., 2019). Goals of therapy include resolution of symptoms and mucosal healing. However, many patients have suboptimal responses to currently available therapies. Therefore, understanding the mechanism of CD is critical to optimizing current treatment strategies.

In recent years, it has been determined that the gut microbiota plays a key role in CD (He et al., 2019). The intestinal flora and the intestinal immune system are always in homeostasis. Breaking this balance can trigger an excessive intestinal immune response and cause the damage to the intestinal mucosal barrier (Nishikawa et al., 2009; Allen-Vercoe and Jobin, 2014). *Fusobacterium nucleatum* (*F. nucleatum*) is a well-known pro-inflammatory bacterium that has been found in many patients with Crohn’s disease (Kostic et al., 2013). Studies have shown that *F. nucleatum* is implicated in CD and that strains isolated from inflamed biopsy tissue from CD patients were significantly more invasive than strains that were isolated from healthy tissue from either CD patients or control patients (Cheung and Bellas, 2007; Han et al., 2010; Strauss et al., 2011). However, the effects and mechanisms of *F. nucleatum* on the CD disease process are not well-defined.

The intact intestinal mucosal barrier can prevent intestinal bacteria, toxins, and antigens from entering immune cells in the lamina propria (Actis et al., 2014). Mucosal healing has been considered the best therapeutic endpoint for CD patients because it is associated with sustained clinical remission followed by a lower incidence of hospitalization and surgery (Malluta et al., 2019). Previous studies have shown that in biofilms, *F. nucleatum* can penetrate the epithelial/basement membrane barrier and invade the collagen matrix after incubation if the bacterial biofilm is incubated in contact with cells in an organotypic cell culture model (Gursoy et al., 2010). *F. nucleatum* can invade into mucosa in patients with acute appendicitis and colorectal cancer (Yu et al., 2016). However, the damage to the intestinal mucosa by *F. nucleatum* has not yet been specifically clarified (Kumar et al., 2016).

Endoplasmic reticulum (ER), as a membrane-bound organelle, plays a crucial role in folding of secreted and membrane proteins (Huang et al., 2019). If the levels of the unfolded and misfolded proteins exceed the processing capacity of the ER, ER stress (ERS) occurs (Li et al., 2019). The ER chaperone protein BIP is a major regulatory factor of ER homeostasis and stress response (Li et al., 2016). Many factors can cause ER homeostasis to be disrupted, including bacterial infection (Ma et al., 2019). Studies have found that microbial infection can trigger ERS, and ERS-activating cells can regulate the expression and activation of ERS-related proapoptotic molecules, ultimately determining whether cells are adaptive or undergo apoptosis (Ma et al., 2019). This response allows pro-inflammatory molecules to be released during the chronic inflammation of the CD, leading to damage to the colon cells, and thereby impairing the integrity of the epithelial barrier. It has been found that the endogenous metabolite acrolein induces ERS, mediates epithelial cell death, leads to impaired intestinal epithelial barrier function and increased permeability, and causes the downregulation and/or redistribution of three representative tight junction proteins (i.e., zonula occludens-1, occludin, and claudin-1) that critically regulate epithelial paracellular permeability (Chen et al., 2017; Odenwald and Turner, 2017). This finding indicates that ERS is closely related to the integrity and function of the intestinal mucosal barrier. However, it is unclear whether *F. nucleatum* can induce intestinal mucosal damage by inducing ERS.

In this study, we investigated whether and how *F. nucleatum* affects the integrity of the epithelial barrier in patients with CD. We examined that the *F. nucleatum* abundance in colon tissue from patients with active CD was increased compared to that in tissues from healthy controls or patients with remitted CD. We then demonstrated that *F. nucleatum* plays a key role in mediating CD development by upregulating caspase activation and recruitment domain 3 (CARD3) and activating the ERS pathway.

## Materials and Methods

### Collection of Clinical Samples

The patient materials used in this study were obtained from Wuhan University People’s Hospital (Hubei, China). All participants provided informed consent, and the project was approved by the institutional review board (approval number: 2018K-C089). Inflamed intestinal biopsies were obtained from CD patients undergoing intestinal endoscopy. Normal tissue biopsies were obtained from healthy controls ranging in age from 15 to 65 years (to match the age of patients with CD) who underwent an endoscopy for colon cancer screening without a prior diagnosis of gastrointestinal illness. CD diagnosis was confirmed in conjunction with clinical and histological criteria. Clinical disease activity was assessed by the Harvey-Bradshaw activity index (HBI) and the colitis activity index. CD patients with an HBI of ≤4 were considered to be in remission, those with ≥5 to have active disease. Exclusion criteria included patients with previous inflammatory bowel disease (IBD) treatment, receiving antibiotics or probiotics in the last 12 weeks, receiving biologicals or immunosuppressants within the past 2 years, history of fecal microbiota transplant (FMT), age <15 years, presentation of other known chronic diseases, and pregnant or breastfeeding. Formalin-fixed, paraffin-embedded CD intestinal tissues were obtained from the pathology archives from June 2016 to June 2018. Clinicopathological data for each patient were obtained from hospital records.

### Bacterial Strains and Cell Lines

The human normal epithelial cell line NCM460 and the FHC cell line ATCC were grown in Dulbecco’s modified Eagle medium (DMEM) supplemented with 10% fetal bovine serum at 37°C and 5% CO_2_. *F. nucleatum* was grown on tryptic soy containing 5% defibrinated sheep blood, under anaerobic conditions (10% H_2_, 5% CO_2_, and 85% N_2_), with a 2-day anaerobic gas filling system at 37°C. *Escherichia coli* strain (Tiangen, China) was cultured in Luria-Bertani (LB) medium for 16 h at 37°C with shaking at 200–220 rpm. The *F. nucleatum* suspension was centrifuged at 2,500 × g for 5 min and was then resuspended in antibiotic-free DMEM before being used to infect normal epithelial cells. *E. coli-*infected cells were used as the control.

### Mice

Five- to six-week-old male C57BL/6J CARD3 knockout (KO, CARD3^-/-^) mice were kindly provided by Dr. Richard Flavell (Howard Hughes Medical Institute, Yale University, New Haven, CT). CARD3 KO mice were backcrossed with C57BL/6 mice for at least six generations to yield CARD3 heterozygous mice. Then, the littermate offspring [card3 KO and wild-type (WT) mice] produced by inbreeding CARD3 heterozygous mice were used for further study. Five- to six-week-old male C57BL/6J WT (CARD3wt) mice were obtained from Nanjing Biomedical Research Institute of Nanjing University (NBRI). Mice were housed and bred in our specific pathogen-free animal facility (Yang et al., 2017). All animal procedures were approved by the Animal Care and Use Committee of Renmin Hospital of Wuhan University, China.

### Induction of Colitis

All mice were fed 2 mg/ml streptomycin for 3 days in their drinking water to ensure consistency of the conventional microbiota and promote *F. nucleatum* colonization. Mice were administered a daily dose of *F. nucleatum* cells [10^9^ colony-forming units (CFUs)/ml] resuspended in phosphate-buffered solution (PBS) or PBS alone for 2 weeks. To induce colitis, 3% (wt/vol) dextran sulfate sodium (DSS) (wt. 36–50 kDa; MP Biomedicals) was added to the drinking water of the mice (Danese et al., 2007). Animal weight, water/food consumption, morbidity, stool consistency, and the presence of large amounts of blood in the feces or anus were measured or observed once daily. On the seventh day after induction with DSS, animals were quickly euthanized by inhalation of CO_2_, the colon and cecum were quickly separated, the colon was photographed and used for length measurement, and feces and blood were gently removed with 4°C PBS. A small segment of the colon was fixed in paraformaldehyde for histological staining (H&E) and fluorescence *in situ* hybridization (FISH), and another portion of the tissue was immediately frozen in liquid nitrogen for PCR or Western blot (WB) analysis.

### Inhibition of Endoplasmic Reticulum Stress With 4-Phenyl Butyric Acid

In the 4-phenyl butyric acid (4-PBA) treatment study, mice were injected intraperitoneally with 4-PBA every 3 days at a dose of 100 μg per mouse, and *F. nucleatum* (10^9^ CFU/ml) resuspended in PBS were administered intragastrically 1 h after 4-PBA injection (Wu et al., 2018). And these mice were treated with DSS and continued for another week. Similarly, NCM460 cells were treated with 4-PBA and were then infected with *F. nucleatum* (MOI = 100) 1 h later.

### RNA Extraction and Real-Time PCR

Total RNA was extracted using TRIpure Total RNA Extraction Reagent (ELK Biotechnology), and real-time PCR with three replicate wells per sample was performed on a StepOne™ Real-Time PCR machine (Life Technologies), using an EnTurbo™ SYBR Green PCR SuperMix kit (ELK Biotechnology, EQ001). Real-time quantitative PCR was performed in triplicate. The Ct values obtained from different samples were compared using the ΔΔCt method. Glyceraldehyde 3-phosphate dehydrogenase (GAPDH) served as the internal reference gene.

### Fluorescence *in Situ* Hybridization

Microbial FISH was performed as described (Yu et al., 2016). Five-micrometer-thick sections were prepared and hybridized following the manufacturer’s instructions (FOCOFISH, Guangzhou, China). The sequence of the “universal bacterial” probe (EUB338; Cy3 labeled) was 5′-GCT GCC TCC CGT AGG AGT-3′. The sequence of the *F. nucleatum*-targeted probe [FUS664; fluorescein isothiocyanate (FITC) labeled] was 5′-CTT GTA GTT CCG C(C/T) TAC CTC-3′. Slides were examined using a microscope (BX53F; Olympus, Tokyo, Japan). Five random 200× magnification fields per sample were evaluated by three observers blinded to the experimental protocol, and the average number of bacteria per field was calculated. We defined negative, low, or high abundance of *F. nucleatum* as those cases with <5, between 5 and 20, and >20 visualized FUS664 probes per field on average, respectively. Other bacteria were noted as positive with >5 bacteria per field with EUB 338 probe but negative with FUS664 probe.

### High-Throughput Sequencing

DNA or RNA was sent to Adaptive Biotechnology (HuaDa, China) for sequencing. The DNA sequence data have been deposited in the NCBI Sequence Read Archive (SRA) database (accession number: PRJNA541040).

### Cell Transfection

siRNA targeting the human CARD3 gene (siCARD3) and nontargeting siRNAs (control siRNAs) were purchased from Guangzhou RiboBio Co., Ltd. (Guangzhou, China). Cells were cultured and transfected with siRNAs according to the supplier’s instructions. Lipid-based transfections were achieved with Lipofectamine 6000 (Beyotime, China) according to the manufacturer’s protocol. Cells were incubated with the siRNA complex for 72 h, and protein was extracted for assessing transfection efficiency by WB.

### Immunohistochemistry and Western Blotting

Immunohistochemistry (IHC) was performed using an UltraSensitive™ SP (mouse/rabbit) IHC kit (Maxib, Fuzhou, China) according to the manufacturer’s instructions. For WB, primary antibodies against the following targets were used: CARD3 (CST), BIP (CST), XBP1 (Abeam), ZO-1 (Abeam), occludin (Abeam), and GAPDH (Bioworld).

### Statistical Analyses

Statistical analysis was performed using GraphPad Prism software version 8.0. Data are expressed as the means ± SDs. Normally distributed data were analyzed by Student’s *t* test. Differences among multiple groups were evaluated for significance using one-way ANOVA combined with Bonferroni’s *post hoc* test. Statistical significance was defined as *P* < 0.05.

## Results

### *F. nucleatum* Is Abundant in Crohn’s Disease Tissues and Is Linked to Disease Severity

**Figure 1:**
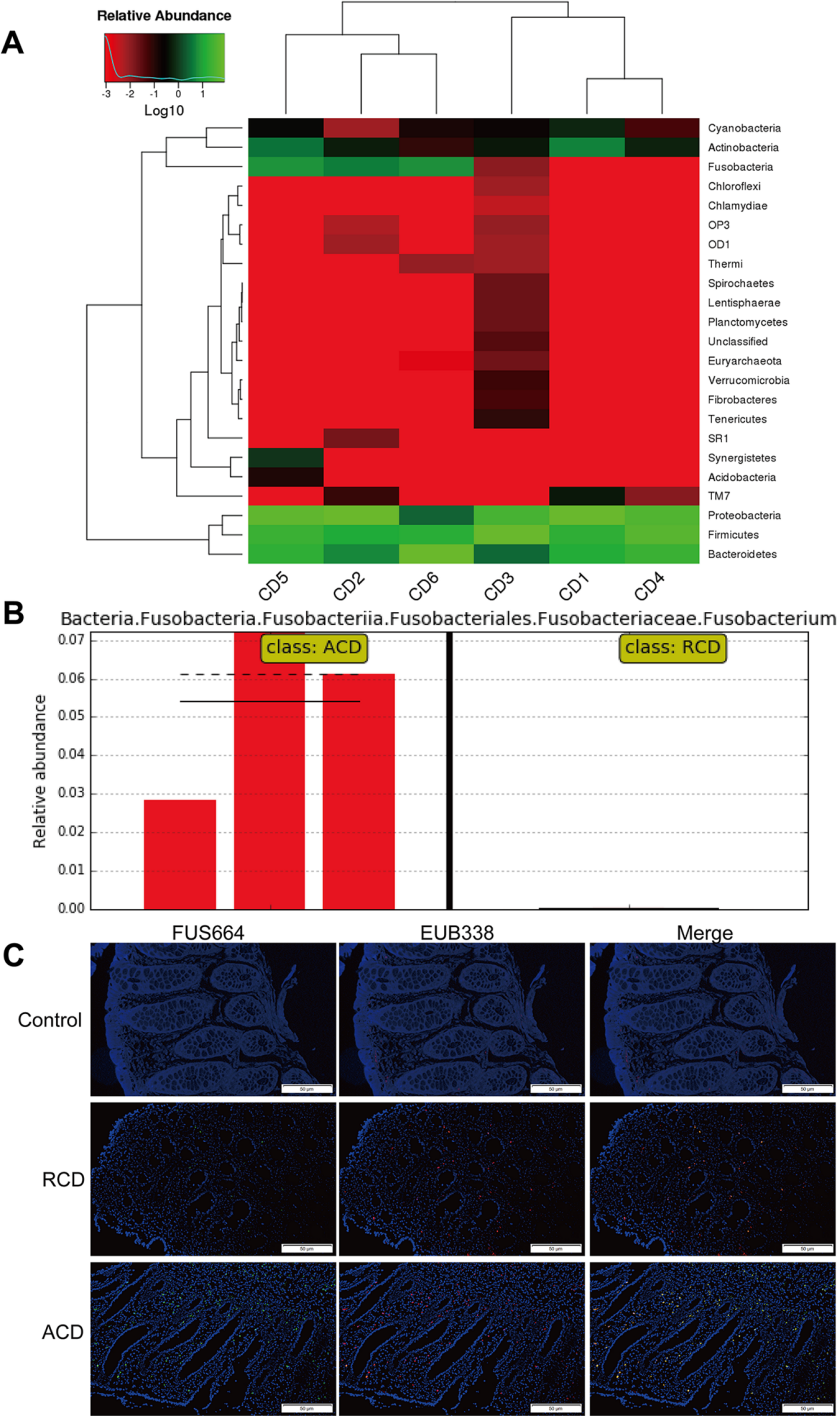
*Fusobacterium nucleatum* is associated with Crohn’s disease (CD) activity. **(A)** Hierarchically clustered heat map representing bacterial taxa (genus level) in six CD tissues from patients [active CD (ACD) = 3, remitted CD (RCD) = 3] by 16S rDNA sequencing. The relative percentages of bacteria are indicated by varying color intensities. Species with an abundance of less than 0.5% in all samples were classified as “Others.” ACD: CD2, CD5, CD6; RCD: CD1, CD3, CD4. **(B)** The LEfSe algorithm was used to identify *Fusobacterium* in ACD and RCD tissues from patients. **(C)** Representative fluorescence *in situ* hybridization (FISH) images to assess the *F. nucleatum* abundance in ACD (n = 22), RCD (n = 11), and healthy tissues (n = 10). EUB338 (red) is a Cy3-conjugated universal bacterial oligonucleotide probe; FUS664 (green) is a fluorescein isothiocyanate (FITC)-conjugated *F. nucleatum-*specific oligonucleotide probe. Magnification, 200×. The sequence of the FITC-labeled Fn-targeted probe, FUS664, was: 5′- CTT GTA GTT CCG C(C/T) TAC CTC -3′.

Previous studies have shown that the invasive potential of gut mucosa-derived *F. nucleatum* positively correlated with CD status of the host (Zhou et al., 2016; Dinakaran et al., 2018). To verify the potential relationship between gut microbiota alterations and CD, we compared three inflamed tissues from patients with active CD (ACD) and three inflamed tissues from patients with remitted CD (RCD) by HiSeq 2500 sequencing. We also found that *F. nucleatum* was enriched in ACD tissues (*P* < 0.05; **Figures 1A, B**). We further examined the abundance of invasive *F. nucleatum* in 33 CD tissues (RCD = 11; ACD = 22) from patients and in 10 normal tissues using FISH. *F. nucleatum* was detected in a significantly higher percentage of CD tissues (41.21%) than control normal tissues (10%; *P* < 0.05; **Figure 1C**). Moreover, *F. nucleatum* was detected in a significantly higher percentage of ACD tissues (46.71%) than RCD tissues (13.52%; *P* < 0.05; **Figure 1C**). This result suggests that invasive *F. nucleatum* is present in CD tissues. We then evaluated the relationship between the abundance of *F. nucleatum* and clinicopathological features as shown in **Table 1**. The abundance of *F. nucleatum* was positively associated with the clinical course, clinical activity, and refractory behavior (*P* < 0.05). Thus, these data defined the potential value of the abundance of *F. nucleatum* in predicting CD activity.

**Table 1:** Clinicopathologic characteristics in *Fusobacterium. nucleatum*-negative vs. *F. nucleatum*-positive CD.

Characteristics		*F. nucleatum*-negative (n = 15)	*F. nucleatum*-positive (n = 18)	*P* value^a^
Gender				
Male	6		12	0.126
Female	9		6	
Age				
≤16	0		1	0.505
≤40	11		11	
>40	4		7	
Clinical course				
Moderate	7		13	0.035*
Severe	0		2	
Remission	8		3	
Location				
L1	5		7	0.731
L2	4		3	
L3	6		7	
L4	0		1	
Behavior				
B1	7		5	0.495
B2	5		7	
B3	3		6	
Perianal disease				
Yes	0		4	0.405
No	0		14	
Surgery				
Yes	3		11	0.017*
No	12		7	

a Chi-square test, *P < 0.05.

CD, Crohn's disease.

### *F. nucleatum* Destroys Epithelial Barrier Function *In Vitro* and *In Vivo*

**Figure 2:**
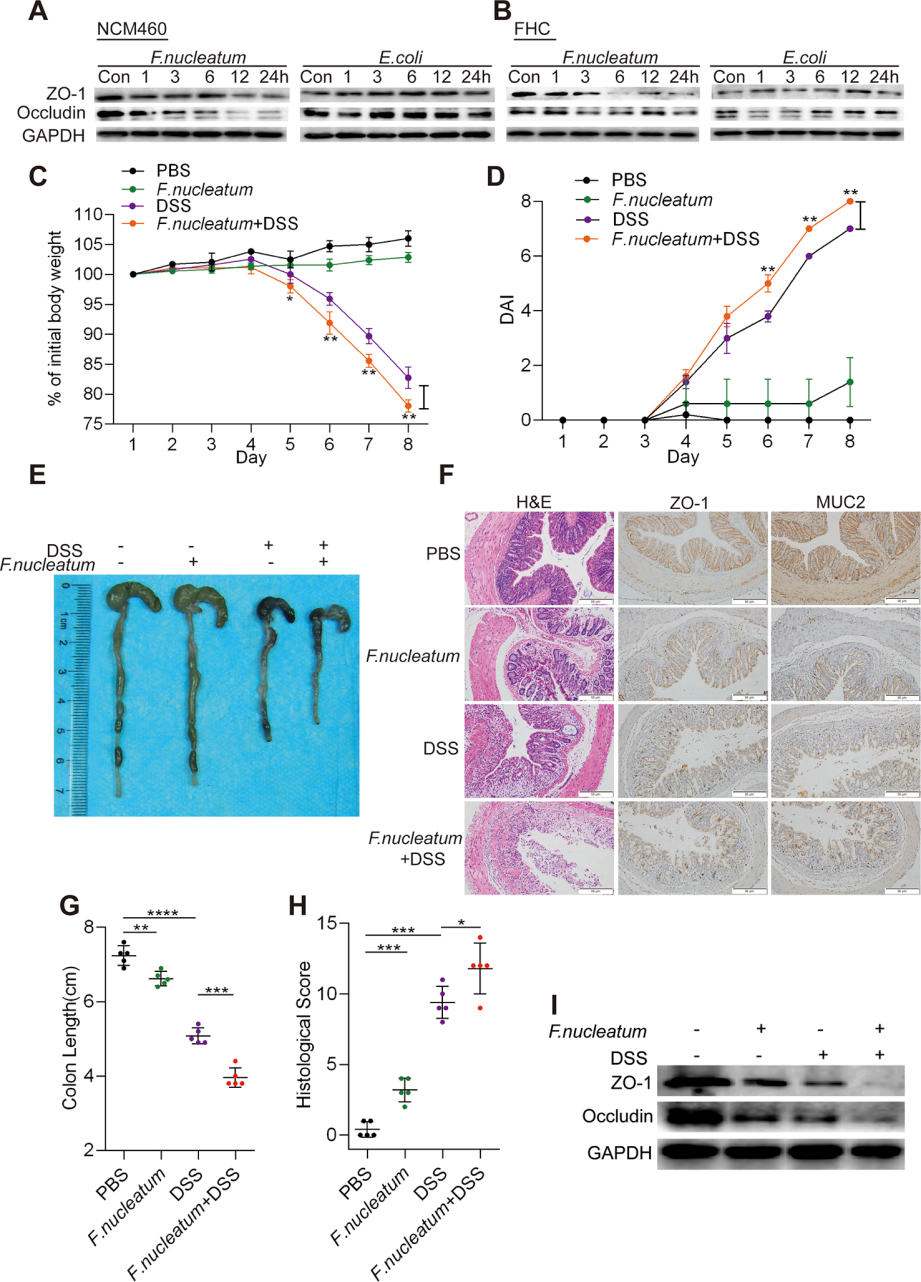
*Fusobacterium nucleatum* destroys epithelial barrier function *in vitro* and *in vivo.* **(A, B)** Western blotting was performed to measure the expression of ZO-1 and occludin in NCM460 cells **(A)** and FHC cells **(B)** cocultured with *F. nucleatum*, *Escherichia coli*, or phosphate-buffered solution (PBS) (Control, Con). **(C, D)** Mice (n = 5 per group) were administered *F. nucleatum* or PBS for 2 weeks and treated with 3% dextran sulfate sodium (DSS) for 7 days. Colitis induction was evaluated by body weight loss **(C)** and the disease activity index (DAI) **(D)**. (*p < 0.05, **p < 0.01, and ***p < 0.001; one-way ANOVA combined with Bonferroni’s *post hoc* test; the error bars indicate the SDs). **(E–G)** Representative colon morphology and length in the mice are shown in panel **(E)** and quantified in panel **(G)**. The sections used for HE, MUC2, and ZO1 staining were from the same mouse in the same group and three sections of the same tissue were stained separately. Representative images of histological analyses are shown in panel **(F)** and quantified in panel **(H)**. Representative images of MUC2 and ZO-1 expression are shown in panel **(F)** (*p < 0.05, **p < 0.01, and ***p < 0.001; unpaired Student’s *t* test; the error bars indicate the SDs; 200× magnification). **(I)** Western blotting was performed to measure ZO-1 and occludin expression in mouse tissues.

We hypothesized that *F. nucleatum* infection may enhance the breakdown of intestinal epithelial barrier function in CD. To test this hypothesis, we incubated NCM460 cells (**Figure 2A**, **Supplementary Figures S1 A–D**) and FHC cells (**Figure 2B**, **Supplementary Figures S1 E–H**) with *F. nucleatum* (ATCC10953), DH5α, or PBS (Control, Con). Compared to DH5α and PBS treatment, *F. nucleatum* treatment downregulated the levels of ZO-1 and occludin in a time-dependent manner, suggesting that *F. nucleatum* may destroy epithelial barrier formation by interfering with tight junction protein expression at the protein level.

To investigate the roles of *F. nucleatum* in the development of colitis, we used a DSS-induced colitis model. Mice treated with *F. nucleatum* + DSS exhibited more severe colitis symptoms, including rapid weight loss (**Figure 2C**) and higher disease activity index (DAI) values (**Figure 2D**), than mice treated with DSS or *F. nucleatum* alone. The colon length was measured to determine the extent of colonic injury, and we found that the colons of *F. nucleatum* + DSS group mice were clearly shorter than those of DSS group mice (**Figures 2E, G**). Additionally, *F. nucleatum* enhanced epithelial damage, including mucosal erosion, crypt loss, and lymphocyte infiltration (**Figure 2F**). Consistent with these observations, histological assessment of the colons revealed a significantly higher histological score (HS) (**Figure 2H**) and more severe disease and disruption of mucosal structures in *F. nucleatum* + DSS-treated mice than in DSS-treated mice. *F. nucleatum*-treated mice exhibited a mild inflammatory phenotype (**Figures 2C–H**), suggesting that *F. nucleatum* may exacerbate the clinical and histological features of DSS-induced colitis. The high abundance of *F. nucleatum* was often accompanied by low levels of ZO-1 and MUC2 compared with those in normal tissues (PBS group) (*P* < 0.05; **Figure 2F**). In addition, the WB results showed lower levels of ZO-1 and occludin in colitis tissues from *F. nucleatum* + DSS-treated mice than in tissues from mice in the other groups (**Figure 2I**, **Supplementary Figures S1I, J**). Taken together, these results indicate that *F. nucleatum* possibly disrupt the mucosal barrier *in vivo* and *in vitro*.

### *F. nucleatum* Damages the Mucosal Barrier *via* ER Signaling in Intestinal Epithelial Cells

**Figure 3:**
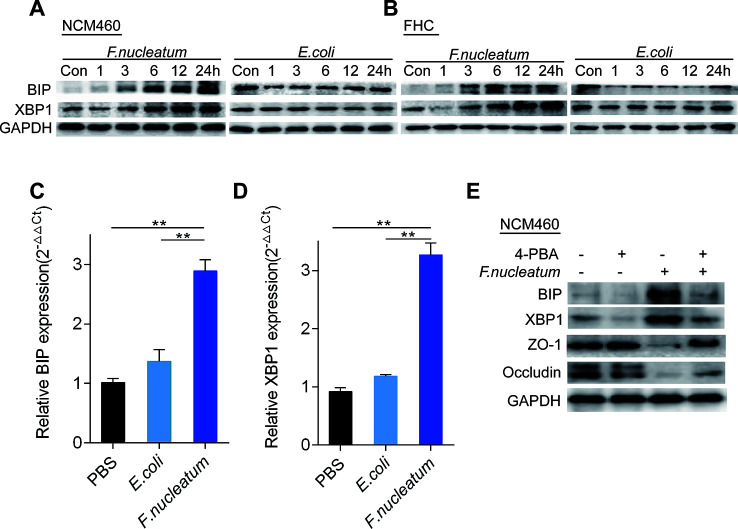
*Fusobacterium nucleatum* activates the endoplasmic reticulum (ER) pathway and damages mucosal barrier-associated proteins *via* ER signaling in intestinal epithelial cell lines. **(A, B)** Western blotting was performed to measure BIP and XBP1 expression in NCM460 cells **(A)** and FHC cells **(B)** cocultured with *F. nucleatum*, *Escherichia coli*, or phosphate-buffered solution (PBS) (Control, Con). **(C, D)** The mRNA expression of BIP **(C)** and XBP1 **(D)** was measured in NCM460 cells cocultured with *F. nucleatum, E. coli*, or PBS (*P < 0.05, **P < 0.01; unpaired Student’s t test; the error bars indicate the SDs). **(E)** Western blotting was performed to measure BIP, XBP1, ZO-1, and occludin expression in NCM460 cells cocultured with *F. nucleatum*, 4-phenyl butyric acid (4-PBA), or both.

To examine whether the ER signaling pathway could be activated by *F. nucleatum* infection, we measured the expression levels of BIP and XBP1 using WB. Compared to DH5α and PBS (Control, Con), *F. nucleatum* treatment upregulated BIP and XBP1 expression in NCM460 cells (**Figure 3A**, **Supplementary Figures S1 K–N**) and FHC cells (**Figure 3**, **Supplementary Figures S2 A–D**) in a time-dependent manner. Additionally, compared to DH5α and PBS treatment, *F. nucleatum* increased the mRNA levels of BIP (**Figure 3C**) and XBP1 (**Figure 3D**) in NCM460 cells (*P* < 0.05), suggesting that *F. nucleatum* may activate the ER pathway in intestinal epithelial cells.

We hypothesized that *F. nucleatum* destroys the intestinal mucosal barrier through the ER pathway. To test this hypothesis, we treated NCM460 cells with 4-PBA (10 μg/ml) or PBS 1 h before *F. nucleatum* (MOI = 100) treatment. Many investigations indicate that 4-PBA acts as a chemical chaperone that attenuates ERS in different cell types (Kolb et al., 2015). Our WB analysis showed that pretreatment with 4-PBA downregulated BIP and XBP1 expression, and blockade of the ER pathway by 4-PBA significantly attenuated the *F. nucleatum*-mediated decrease in ZO-1 and occludin (*P* < 0.05; **Figure 3E**, **Supplementary Figures S2 E–H**). These data indicate that *F. nucleatum* potentially damages the intestinal mucosal barrier *via* ER signaling in epithelial cells.

### *F. nucleatum* Facilitates Intestinal Mucosal Barrier Destruction Through the ER Pathway in DSS-Induced Mice

**Figure 4:**
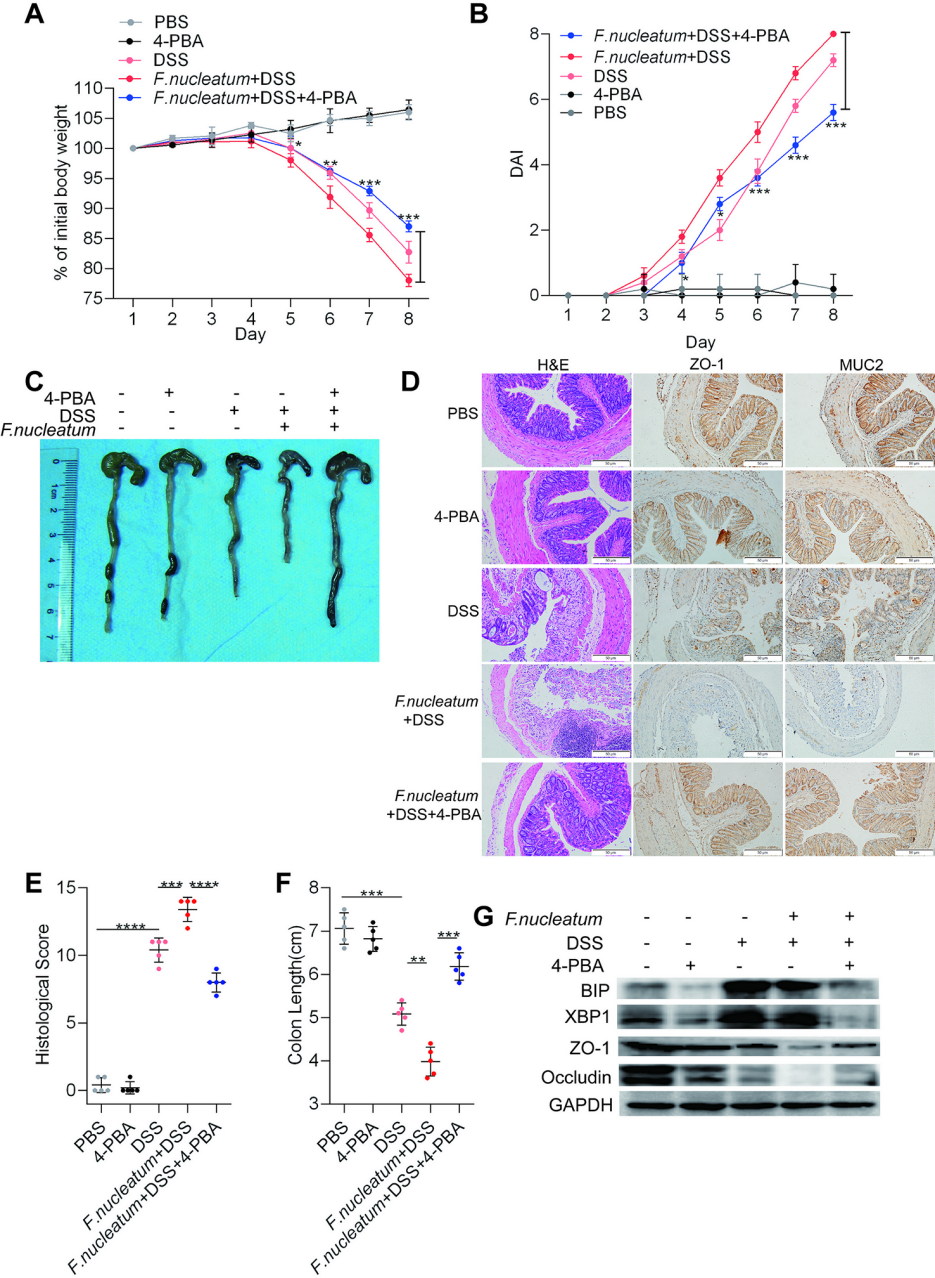
*Fusobacterium nucleatum* facilitates intestinal mucosal barrier destruction through the endoplasmic reticulum (ER) pathway in dextran sulfate sodium (DSS)-induced mice. **(A–F)** Mice (n = 5 per group) were given 4-phenyl butyric acid (4-PBA) (100 μg per mouse every 3 days) and treated with *F. nucleatum* or phosphate-buffered solution (PBS) for 2 weeks. Then, these mice were administered DSS along with continued 4-PBA treatment for an additional 7 days. Colitis induction was evaluated by body weight loss **(A)** and the disease activity index (DAI) **(B)**. (*P < 0.05, **P < 0.01, and ***P < 0.001; one-way ANOVA combined with Bonferroni’s *post hoc* test; the error bars indicate the SDs). Representative colon morphology and length in the mice are shown in panel **(C)** and quantified in panel **(F)**. Representative images of histological analyses are shown in panel **(D)** and quantified in panel **(E)** (*P < 0.05, **P < 0.01, ***P < 0.001, and ****P < 0.0001; unpaired Student’s *t* test; the error bars indicate the SDs; 200× magnification). **(G)** Western blotting was performed to measure the levels of BIP, XBP1, ZO-1, and occludin in colon tissues from mice.

To assess whether the ER pathway contributes to *F. nucleatum*-induced colitis, 4-PBA was administered to mice with DSS-induced colitis. Mice treated with *F. nucleatum* + DSS + 4-PBA exhibited a slower decline in body weight (*P* < 0.05; **Figure 4A**), a lower DAI (*P* < 0.01; **Figure 4B**), a significantly lower HS (*P* < 0.01; **Figure 4E**), and milder colitis (**Figure 4D**) than mice treated with *F. nucleatum* + DSS. DSS-induced or *F. nucleatum* + DSS-induced colon shortening was mitigated in mice administered 4-PBA (*P* < 0.01; **Figures 4C, F**). Moreover, WB showed that inflamed tissues from *F. nucleatum* + DSS + 4-PBA-treated mice exhibited lower levels of BIP and XBP1 and higher levels of ZO-1 and occludin than tissues from *F. nucleatum* + DSS-treated mice (**Figure 4G**, **Supplementary Figures S2 I–L**). Collectively, these data suggest that *F. nucleatum* possibly destroy the intestinal mucosal barrier at least in part *via* the ER pathway in mice.

### *F. nucleatum* Upregulates CARD3 Expression

**Figure 5:**
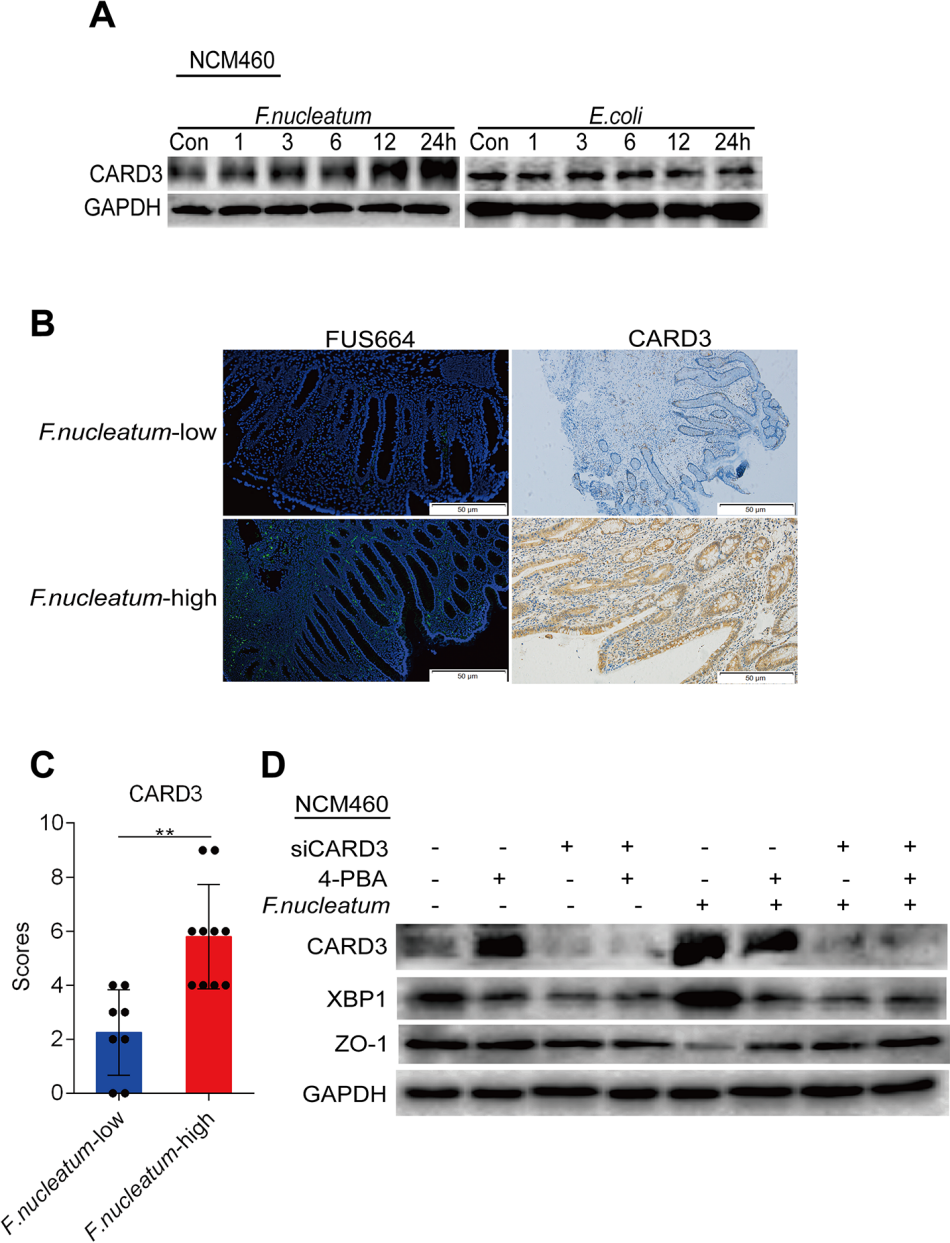
*Fusobacterium nucleatum* is associated with caspase activation and recruitment domain 3 (CARD3) expression and activates the endoplasmic reticulum (ER) pathway *via* CARD3 in NCM460 cells. **(A)** Western blotting was performed to measure CARD3 protein expression over time in NCM460 cells cocultured with *F. nucleatum* or *Escherichia coli* over time. **(B, C)** Representative images showing that the abundance of invasive *F. nucleatum* in CD tissues is associated with high expression of CARD3 (**P < 0.01; unpaired Student’s *t* test; the error bars indicate the SDs; 200× magnification). **(D)** Western blotting was performed to measure the protein levels of CARD3, XBP1, and ZO-1 in NCM460 cells transfected with nontargeting siRNAs (NC), 4-phenyl butyric acid (4-PBA), or siCARD3 and infected with *F. nucleatum*.

CARD3 is known for its role in inflammation (Irving et al., 2014). Previous studies have found that genetic loss of CARD3 is protective against colitis through decreased epithelial cell apoptosis and consequent enhancement of intestinal epithelial barrier function (Yu et al., 2015). We hypothesized that CARD3 may be a target of *F. nucleatum* in disease development. To verify this hypothesis, we treated NCM460 cells with *F. nucleatum* (ATCC10953), DH5α, or PBS (Control, Con). Compared to DH5α and PBS treatment, *F. nucleatum* treatment increased the levels of CARD3 in a time-dependent manner (**Figure 5A**), suggesting that *F. nucleatum* may regulate CARD3 expression at the protein level. We next used FISH to visualize the amount of *F. nucleatum*, as well as the expression of CARD3, in inflamed tissues from CD patients. A high abundance of *F. nucleatum* in inflamed colon tissues was often accompanied by a high level of CARD3 expression (*P* < 0.05; **Figures 5B, C**). These data support the hypothesis that CARD3 is probably a downstream target of *F. nucleatum*.

### *F. nucleatum* Modulates the ER Pathway *Via* Card3 Upregulation *In Vitro* and *In Vivo*

The increase in CARD3 expression in *F. nucleatum*-infected NCM460 cells led us to hypothesize that CARD3 may regulate *F. nucleatum*-mediated ER pathway activation. To test this hypothesis, we transfected NCM460 cells with CARD3-targeting siRNA (siCARD3) or nontargeting siRNAs (NC) (**Supplementary Figures S3 A, B**). Knockdown of CARD3 suppressed the *F. nucleatum*-induced increase in the protein levels of XBP1 and the *F. nucleatum*-induced decrease in the expression of ZO-1 expression (*P* < 0.05; **Figure 5D**, **Supplementary Figures S3 C, D**), highlighting the role of CARD3 in *F. nucleatum*-mediated ER pathway activation in NCM460 cells.

**Figure 6:**
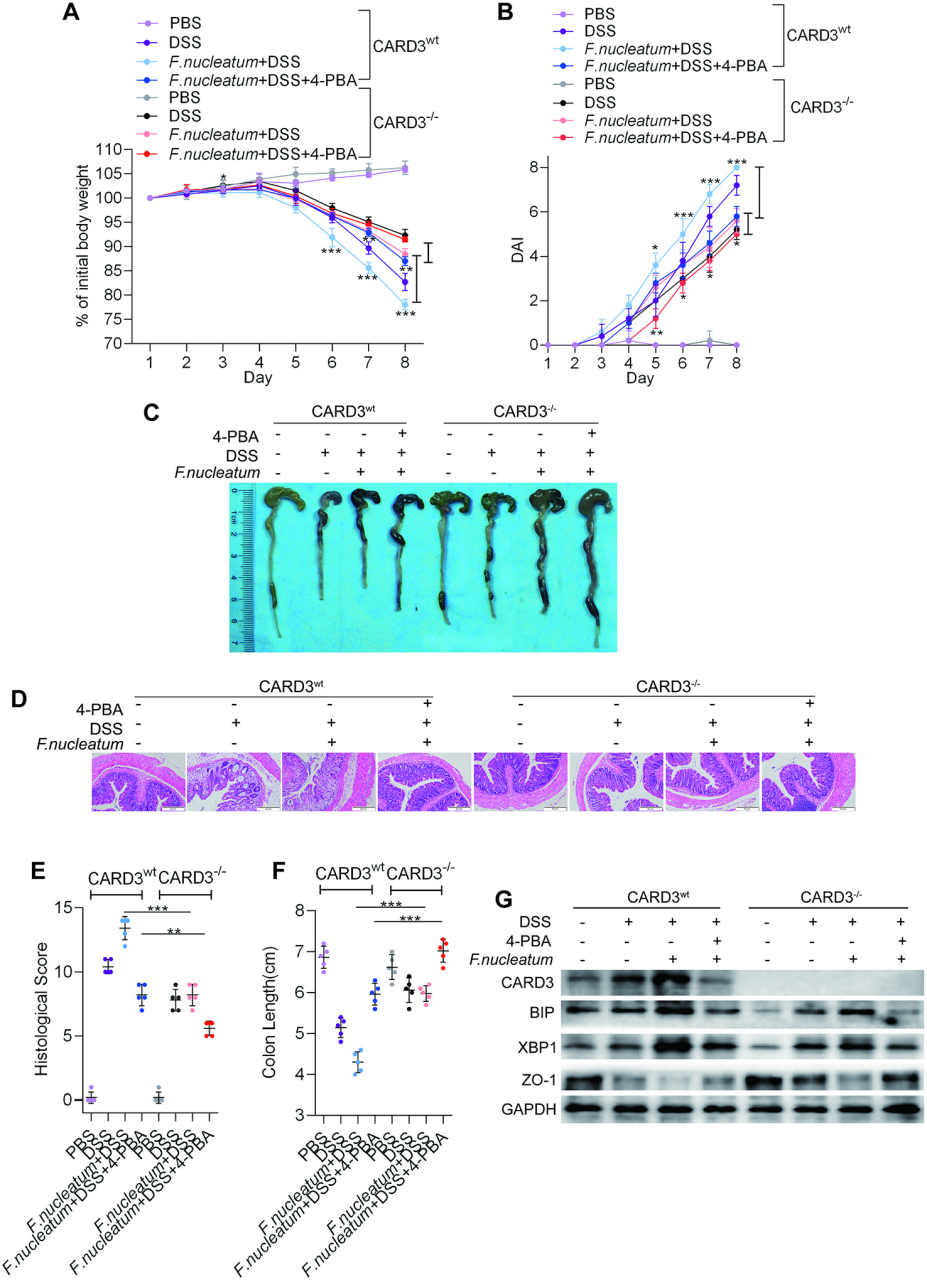
*Fusobacterium nucleatum* activates the endoplasmic reticulum (ER) pathway through the upregulation of caspase activation and recruitment domain 3 (CARD3) in dextran sulfate sodium (DSS)-induced mice. **(A–F)** CARD3^wt^ mice and CARD3^–/–^ mice (n = 5 per group) were administered intraperitoneal injections of 4-phenyl butyric acid (4-PBA) and treated with *F. nucleatum* or phosphate-buffered solution (PBS) for 2 weeks. Then, these mice were administered DSS along with continued 4-PBA treatment for an additional 7 days. Colitis induction was evaluated by body weight loss **(A)** and the disease activity index (DAI) **(B)**. (*P < 0.05, **P < 0.01, and ***P < 0.001; one-way ANOVA combined with Bonferroni’s *post hoc* test; the error bars indicate the SDs). Representative colon morphology and length in the mice are shown in panel **(C)** and quantified in panel **(F)**. Representative images of histological analyses are shown in panel **(D)** and quantified in panel **(E)** (*P < 0.05, **P < 0.01, ***P < 0.001, and ****P < 0.0001; unpaired Student’s *t* test; the error bars indicate the SDs; 200× magnification). **(G)** Western blotting was performed to measure the levels of CARD3, BIP, XBP1, and ZO-1 in colon tissues from mice.

To further confirm our hypothesis that *F. nucleatum* promotes colitis progression through ER activation *via* the upregulation of CARD3 expression, we employed CARD3 knockout (KO, CARD3^–/–^) mice. Both CARD3 WT (CARD3^wt^) and CARD3^–/–^ mice were initially administered *F. nucleatum* and were then subjected to DSS treatment. CARD3^–/–^ mice exhibited slower body weight loss (*P* < 0.01; **Figure 6A**), a lower DAI (*P* < 0.05; **Figure 6B**), a significantly lower HS (*P* < 0.05; **Figure 6C, F**), a shorter colon length (*P* < 0.05; **Figures 6C–F**), and milder colitis (**Figure 6D**) than CARD3^wt^ mice. After knockout of CARD3, treatment with 4-PBA or both, DSS-induced cecal edema, colon shortening and colitis were significantly suppressed (*P* < 0.05; **Figures 6C–F**). Within lesions, the levels of BIP and XBP1 were decreased, and the expression of ZO-1 was increased in CARD3^–/–^ mice (**Figure 6G**, **Supplementary Figures S3 F–I**). Taken together, our data show that CARD3^–/–^ mice are less susceptible to gut inflammation and *F. nucleatum* infection than CARD3^wt^ mice, suggesting a role for CARD3 in the regulation of *F. nucleatum*-induced intestinal mucosal barrier destruction.

## Discussion

Recent studies have shown that *F. nucleatum* in the gut microbiota is involved in the development of colitis. Infection with *F. nucleatum* is often observed in patients with CD (Allen-Vercoe et al., 2011; Kaakoush et al., 2012; Purcell et al., 2018). Similarly, our high-throughput sequencing has demonstrated an increased abundance of *F. nucleatum* in colon tissues from CD patients. *F. nucleatum* infiltrated the epithelium and mucosa and is associated with the clinical activity and severity of colitis (Swidsinski et al., 2011; Strauss et al., 2011). We confirmed that *F. nucleatum* invades CD tissue and is associated with the degree of clinical activity and refractory behavior of CD. In addition, in DSS-induced colitis models, we used oral administration of *F. nucleatum* before the induction of acute colitis with DSS to initially assess the precise role of *F. nucleatum* in IBD. Through a combination of biological methods, *in vivo* models, and clinical studies, we demonstrated that *F. nucleatum* was enriched in CD tissues from patients and exacerbated colonic inflammation during CD development.

It has been confirmed that *F. nucleatum* contributes to the carcinogenesis of colorectal cancer by inducing inflammation and inhibiting host immunity (Feng et al., 2019; Wu et al., 2019). There is evidence that *F. nucleatum* induces the production of microbial peptides that upregulate the expression of proinflammatory cytokines and tumor necrosis factor and disrupt homeostasis and host defense barriers (Krisanaprakornkit et al., 2000; Repass et al., 2018). An *F. nucleatum* strain isolated from IBD patients showed upregulation of monocyte chemoattractant protein (MCP)-1 and tumor necrosis factor (TNF)-α in an experimental model (McCoy et al., 2013). However, it is unclear how *F. nucleatum* mediates intestinal inflammation during the development of CD. We examined whether *F. nucleatum* induced the downregulation of membrane-associated proteins (ZO-1 and occludin), which are markers of intestinal mucosal barrier function. We found that *F. nucleatum* infection was negatively correlated with the expression of intestinal mucosal barrier-associated proteins (ZO-1 and occludin) in human epithelial cell lines and human colon tissues and significantly downregulated the expression of intestinal mucosal barrier proteins in a mouse model of DSS-induced colitis. *F. nucleatum* infection can disrupt intestinal mucosal function and has the potential contribution to induce associated colitis.

Next, we investigated the mechanism by which *F. nucleatum* causes intestinal mucosal barrier damage. Studies have shown that when ERS occurs, cells initiate a caspase12-dependent apoptosis program, which triggers the destruction of the intestinal mucosal barrier (Ge et al., 2019). We found that *F. nucleatum* infection significantly increased the expression of ERS-related proteins (BIP and XBP1), which are key regulators of ERS, *in vivo and in vitro* but that by inhibiting ERS with 4-PBA, the intestinal mucosal barrier function and the severity of colitis were significantly mitigated. Therefore, we concluded that *F. nucleatum* may induce intestinal mucosal damage partly by activating the ERS pathway to mediate CD, and colitis may be relieved under the treatment of 4-PBA. The majority of investigations suggest that 4-PBA acts as a chemical chaperone that attenuates ERS in different cell types (Kolb et al., 2015). But whether 4-PBA can act *via* different routes not just as an ERS inhibitor remains to be verified.

In addition, we investigated the mechanism by which *F. nucleatum* mediates the activation of the ERS pathway. Studies have reported that microbes can induce a CARD-dependent inflammatory response in epithelial cells (McCoy et al., 2013; Wang et al., 2013; Li et al., 2015). Previous studies have found that genetic loss of CARD3 is protective against colitis through decreased epithelial cell apoptosis and consequent enhancement of intestinal epithelial barrier function (Tigno-Aranjuez et al., 2014; Yu et al., 2015). Our previous report has found that the expression of NOD2 was upregulated in intestinal epithelial cells infected with *F. nucleatum* (Chen et al., 2019). Thus, we choose CARD3 as the downstream molecule of *F. nucleatum*. In this study, we found that CARD3 expression in CD patients was higher than that in healthy controls and that the abundance of *F. nucleatum* was positively correlated with the expression of CARD3. Some studies have shown that CARD3 is a nucleotide oligomerization domain (NOD)-independent nodal point of gut inflammation (Panda and Gekara, 2018). Decreased expression of NOD1/2 and interaction between NOD1/2 and CARD3 can decrease the severity of ERS (Zhang et al., 2016). It is clear that NOD1/2 and CARD3 are important mediators of ERS-induced inflammation in mouse and human cells (Keestra-Gounder et al., 2016). In the present study, we demonstrated that knockdown or knockout of the CARD3 gene can alleviate the extent of *F. nucleatum*-associated colitis and mitigate ERS. We demonstrated for the first time that *F. nucleatum* activates ERS and promotes CD development by upregulating CARD3 expression. However, we did not explore the mechanism by which *F. nucleatum* regulates CARD3, and whether other ERS or CARD3 inhibitors can rescue the CD phenotype induced by *F. nucleatum* + DSS remains to be verified.

From a clinical perspective, since the abundance of *F. nucleatum* is related to the risk of CD activity, measuring the *F. nucleatum* abundance may be an effective method for predicting disease activity in patients. In addition, our data raise an important clinical question: Should CD patients with a high abundance of *F. nucleatum* be treated with conventional treatments against *F. nucleatum* and/or with ERS or CARD3 inhibitors? Our findings support this view. Therefore, it is important to determine the abundance and related pathways of *F. nucleatum* and to differentially manage patients with different abundances of *F. nucleatum*.

Overall, our results demonstrate that *F. nucleatum* promotes the development of CD by regulating the molecular mechanisms involving CARD3. Moreover, the clinical information that we collected from CD patients also indicated that *F. nucleatum* and CARD3 are risk factors for a high degree of disease activity in CD patients. Our research provides new evidence demonstrating the pro-inflammatory effects of *F. nucleatum* in CD and offers new approaches to the assessment of microbial populations and genetic alterations for the treatment and prevention of CD.

## Data Availability Statement

The datasets generated for this study can be found in the NCBI Sequence Read Archive (SRA) using the accession number PRJNA541040.

## Ethics Statement

All participants provided written informed consent, and the project was approved by the Institutional Review Board (Approval number: 2018K-C089). The animal study was reviewed and approved by the Institutional Review Board of Renmin Hospital of Wuhan University, China.

## Author Contributions

Study conception and design: WD, PC, YYC; Specimen provision: NZ, XG; Acquisition of clinical data: PC, YYC, YC, XG; Data analysis and interpretation and statistical analysis: PC, YYC, YC, XG; Animal experiments: PC, YYC, YC, WHS, XG; Manuscript drafting: PC, YYC, WGD.

## Funding

This work was supported by grants from the National Natural Science Foundation of China (No. 81870392, No. 81372551, and No. 81572426) and the Guiding Foundation of Renmin Hospital of Wuhan University (No. RMYD2018Z01).

## Conflict of Interest

The authors declare that the research was conducted in the absence of any commercial or financial relationships that could be construed as a potential conflict of interest.

## Supplementary Material

Supplementary Figure S1
*F. nucleatum* destroys epithelial barrier function *in vitro* and *in vivo* **(A–H)** The protein expression of ZO-1 and Occludin in NCM460 cells **(A–D)** and FHC cells **(E–H)** cocultured with *F. nucleatum*, *E. coli* or PBS (Control, Con) were quantified. **(I, J)** The protein expression of ZO-1 and Occludin expression in mouse tissues were quantified. **(K–N)** The protein expression of BIP and XBP1 in NCM460 cells cocultured with *F. nucleatum*, E. coli or PBS (Control, Con) were quantified. Data are expressed as mean ± SD for three independent experiments. Statistical significance is indicated as follows: **P* < 0.05, ***P* < 0.01, and ****P* < 0.001.

Supplementary Figure S2
*F. nucleatum* activates the ER pathway and damages mucosal barrier-associated proteins via ER signaling *in vitro* and *in vivo* **(A–D)** The protein expression of BIP and XBP1 in FHC cells cocultured with *F. nucleatum*, *E. coli* or PBS (Control, Con) were quantified. **(E–H)** The protein expression of BIP, XBP1, ZO-1 and Occludin in NCM460 cells cocultured with *F. nucleatum*, 4-PBA or both were quantified. **(I–L)** The protein expression of BIP, XBP1, ZO-1 and Occludin in colon tissues from mice were quantified. Data are expressed as mean ± SD for three independent experiments. Statistical significance is indicated as follows: **P* < 0.05, ***P* < 0.01, and ****P* < 0.001.

Supplementary Figure S3
*F. nucleatum* activates the ER pathway through the upregulation of CARD3 *in vitro* and *in vivo* **(A–B)** The protein expression of CARD3 in NCM460 cells cocultured with PBS (Control, Con), NC, si001, si002 or si003 were detected by immunoblot and quantified. **(C–E)** The protein expression of CARD3, XBP1 and ZO-1 in NCM460 cells transfected with NC, 4-PBA or siCARD3 and infected with *F. nucleatum* were quantified. **(F–I)** The protein expression of CARD3, BIP, XBP1 and ZO-1 in colon tissues from mice were quantified. Data are expressed as mean ± SD for three independent experiments. Statistical significance is indicated as follows: **P* < 0.05, ***P* < 0.01, and ****P* < 0.001.
