# Corrigendum: *Fusobacterium nucleatum* Activates Endoplasmic Reticulum Stress to Promote Crohn’s Disease Development via the Upregulation of CARD3 Expression

**DOI:** 10.3389/fphar.2021.672387

**Published:** 2021-05-19

**Authors:** Pan Cao, Yongyu Chen, Xufeng Guo, Yan Chen, Wenhao Su, Na Zhan, Weiguo Dong

**Affiliations:** ^1^ Department of Gastroenterology, Renmin Hospital of Wuhan University, Wuhan, China; ^2^ Key Laboratory of Hubei Province for Digestive System Disease, Wuhan, China; ^3^ Central Laboratory, Renmin Hospital of Wuhan University, Wuhan, China

**Keywords:** F nucleatum, intestinal mucosal barrier, endoplasmic reticulum stress, Crohn’s disease, gene regulation

Xufeng Guo was not included as an author in the published article. The corrected Author Contributions Statement appears below.

In the original article, there was a mistake in Figure 2F as published. The carelessness in combining the images caused the repetition of the images. The corrected Figure 2 appears below.

**FIGURE 2:**
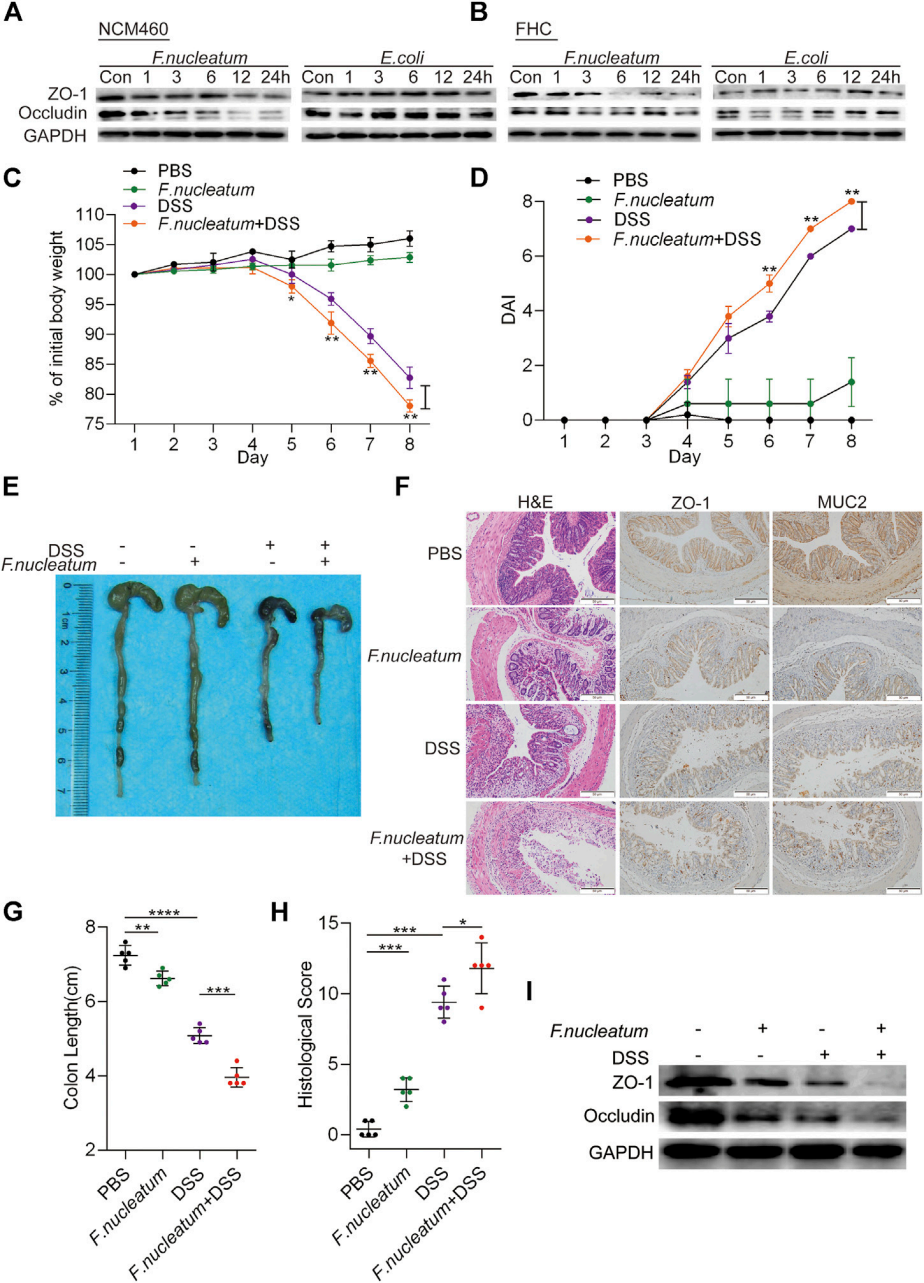
*Fusobacterium nucleatum* destroys epithelial barrier function *in vitro* and *in vivo*. **(A,B)** Western blotting was performed to measure the expression of ZO-1 and occludin in NCM460 cells **(A)** and FHC cells **(B)** cocultured with *F. nucleatum*, *Escherichia coli*, or phosphate-buffered solution (PBS) (Control, Con). **(C,D)** Mice (*n* = 5 per group) were administered *F. nucleatum* or PBS for 2 weeks and treated with 3% dextran sulfate sodium (DSS) for 7 days. Colitis induction was evaluated by body weight loss **(C)** and the disease activity index (DAI) **(D)**. (*p < 0.05, **p < 0.01, and ***p < 0.001; one-way ANOVA combined with Bonferroni’s *post hoc* test; the error bars indicate the SDs). **(E–G)** Representative colon morphology and length in the mice are shown in panel **(E)** and quantified in panel **(G)**. The sections used for HE, MUC2, and ZO1 staining were from the same mouse in the same group and three sections of the same tissue were stained separately. Representative images of histological analyses are shown in panel **(F)** and quantified in panel **(H)**. Representative images of MUC2 and ZO-1 expression are shown in panel **(F)** (*p < 0.05, **p < 0.01, and ***p < 0.001; unpaired Student’s *t* test; the error bars indicate the SDs; 200× magnification). **(I)** Western blotting was performed to measure ZO-1 and occludin expression in mouse tissues.

Furthermore, a brief description was added to the annotations in Figure 2, indicating that these are serial sections from the same animal. The corrected Figure legend appears below.

