# Supplementary figures and images for: Fusobacterium nucleatum Activates Endoplasmic Reticulum Stress to Promote Crohn’s Disease Development via the Upregulation of CARD3 Expression
Source: Front Pharmacol. 2020 Feb 21;11:106. doi: 10.3389/fphar.2020.00106 (PMC7047714; doi:10.3389/fphar.2020.00106)

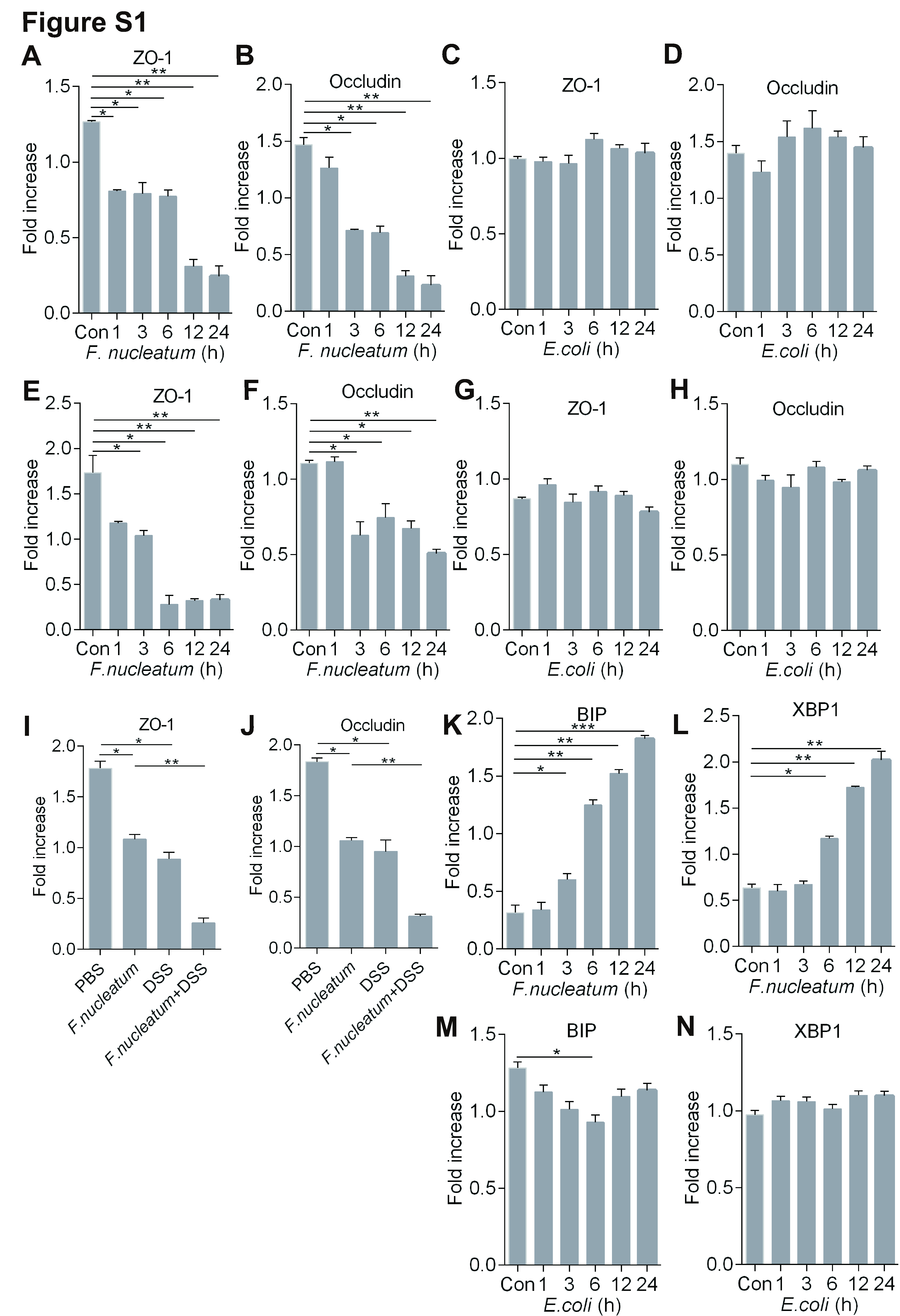

Supplement: Supplementary Figure S1 — F. nucleatum destroys epithelial barrier function in vitro and in vivo (A–H) The protein expression of ZO-1 and Occludin in NCM460 cells (A–D) and FHC cells (E–H) cocultured with F. nucleatum, E. coli or PBS (Control, Con) were quantified. (I, J) The protein expression of ZO-1 and Occludin expression in mouse tissues were quantified. (K–N) The protein expression of BIP and XBP1 in NCM460 cells cocultured with F. nucleatum, E. coli or PBS (Control, Con) were quantified. Data are expressed as mean ± SD for three independent experiments. Statistical significance is indicated as follows: *P < 0.05, **P < 0.01, and ***P < 0.001. [file Image_1.tif]

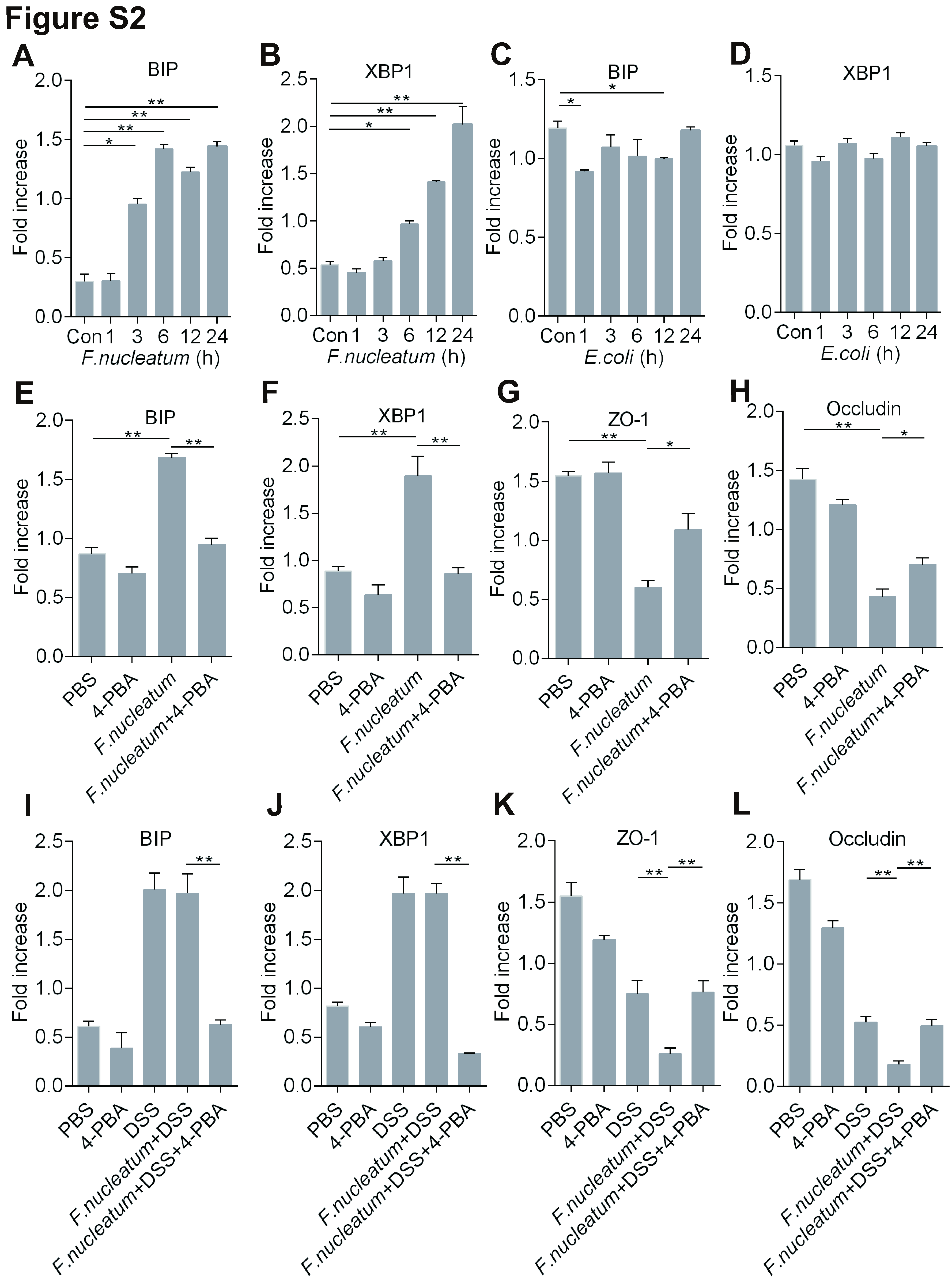

Supplement: Supplementary Figure S2 — F. nucleatum activates the ER pathway and damages mucosal barrier-associated proteins via ER signaling in vitro and in vivo (A–D) The protein expression of BIP and XBP1 in FHC cells cocultured with F. nucleatum, E. coli or PBS (Control, Con) were quantified. (E–H) The protein expression of BIP, XBP1, ZO-1 and Occludin in NCM460 cells cocultured with F. nucleatum, 4-PBA or both were quantified. (I–L) The protein expression of BIP, XBP1, ZO-1 and Occludin in colon tissues from mice were quantified. Data are expressed as mean ± SD for three independent experiments. Statistical significance is indicated as follows: *P < 0.05, **P < 0.01, and ***P < 0.001. [file Image_2.tif]

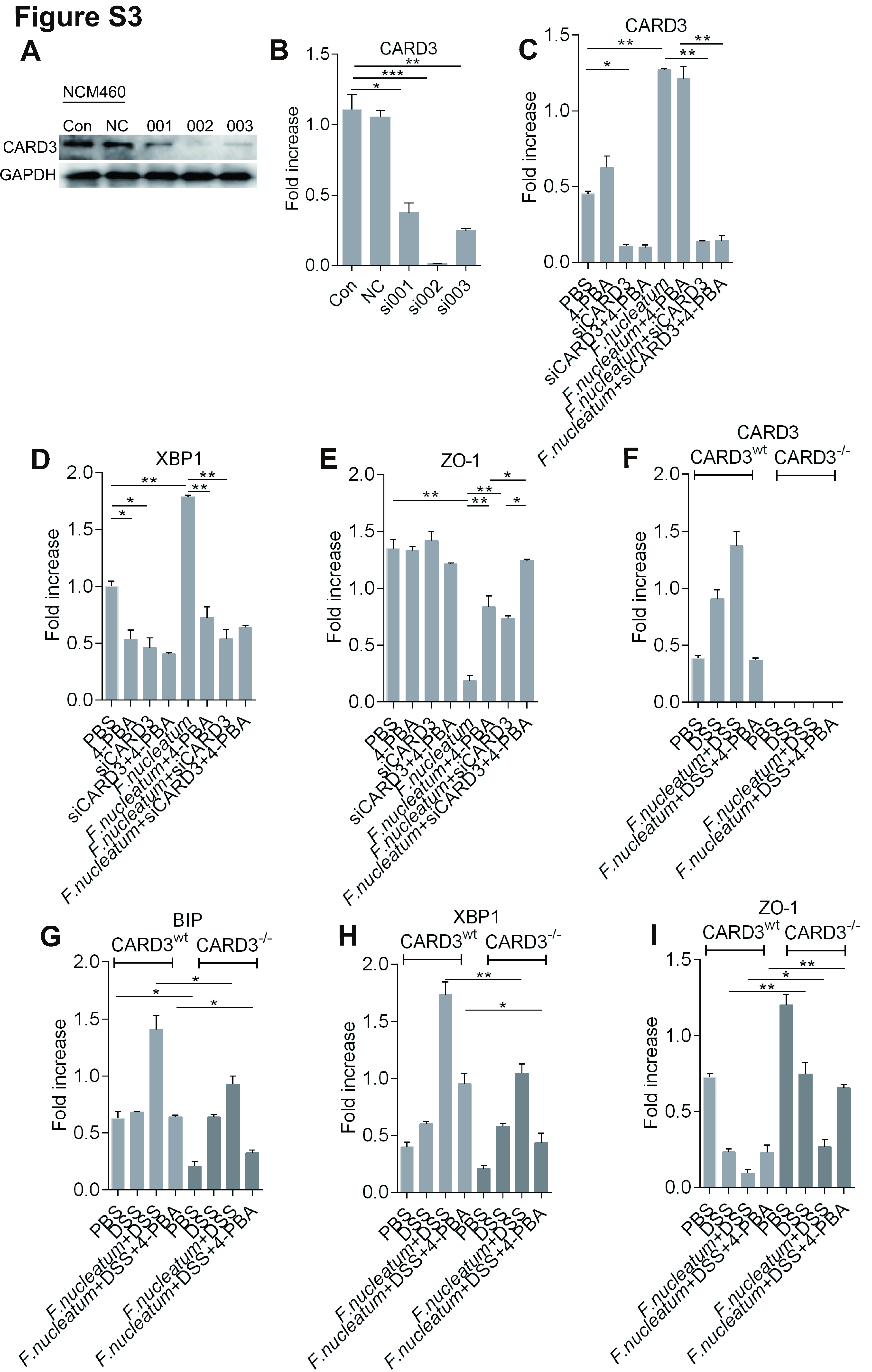

Supplement: Supplementary Figure S3 — F. nucleatum activates the ER pathway through the upregulation of CARD3 in vitro and in vivo (A–B) The protein expression of CARD3 in NCM460 cells cocultured with PBS (Control, Con), NC, si001, si002 or si003 were detected by immunoblot and quantified. (C–E) The protein expression of CARD3, XBP1 and ZO-1 in NCM460 cells transfected with NC, 4-PBA or siCARD3 and infected with F. nucleatum were quantified. (F–I) The protein expression of CARD3, BIP, XBP1 and ZO-1 in colon tissues from mice were quantified. Data are expressed as mean ± SD for three independent experiments. Statistical significance is indicated as follows: *P < 0.05, **P < 0.01, and ***P < 0.001. [file Image_3.tif]
